# Effect of Elevated *p*CO_2_ on Metabolic Responses of Porcelain Crab (*Petrolisthes cinctipes*) Larvae Exposed to Subsequent Salinity Stress

**DOI:** 10.1371/journal.pone.0109167

**Published:** 2014-10-08

**Authors:** Seth H. Miller, Sonia Zarate, Edmund H. Smith, Brian Gaylord, Jessica D. Hosfelt, Tessa M. Hill

**Affiliations:** 1 Bodega Marine Laboratory, University of California Davis, Davis, California, United States of America; 2 Department of Evolution and Ecology, University of California Davis, Davis, California, United States of America; 3 Department of Geology, University of California Davis, Davis, California, United States of America; University of California- Santa Barbara, United States of America

## Abstract

Future climate change is predicted to alter the physical characteristics of oceans and estuaries, including pH, temperature, oxygen, and salinity. Investigating how species react to the influence of such multiple stressors is crucial for assessing how future environmental change will alter marine ecosystems. The timing of multiple stressors can also be important, since in some cases stressors arise simultaneously, while in others they occur in rapid succession. In this study, we investigated the effects of elevated *p*CO_2_ on oxygen consumption by larvae of the intertidal porcelain crab *Petrolisthes cinctipes* when exposed to subsequent salinity stress. Such an exposure mimics how larvae under future acidified conditions will likely experience sudden runoff events such as those that occur seasonally along portions of the west coast of the U.S. and in other temperate systems, or how larvae encounter hypersaline waters when crossing density gradients via directed swimming. We raised larvae in the laboratory under ambient and predicted future *p*CO_2_ levels (385 and 1000 µatm) for 10 days, and then moved them to seawater at ambient *p*CO_2_ but with decreased, ambient, or elevated salinity, to monitor their respiration. While larvae raised under elevated *p*CO_2_ or exposed to stressful salinity conditions alone did not exhibit higher respiration rates than larvae held in ambient conditions, larvae exposed to elevated *p*CO_2_ followed by stressful salinity conditions consumed more oxygen. These results show that even when multiple stressors act sequentially rather than simultaneously, they can retain their capacity to detrimentally affect organisms.

## Introduction

Increasing atmospheric carbon dioxide (CO_2_) is altering Earth’s climate, creating multiple new stressors with which organisms must cope. Predicted changes include more intense and more frequent storms, shifts in the hydrologic cycle, higher air and seawater temperatures, and the ecological challenge of ocean acidification [1], [2], [3], [4], [5]. As the ocean absorbs CO_2_ from the atmosphere, seawater pH decreases, which can make growth and survival more difficult for a wide range of marine and estuarine species [6], [7], [8], [9], [10]. Estuarine and coastal environments may be particularly sensitive to the effects of climate change, as these ecosystems not only experience both terrestrial and marine perturbations, but also encounter an extensive suite of anthropogenic stressors tied to nutrient loading, fishing, pollution, and habitat degradation [11].

Model predictions of future climate change for the northeastern Pacific Ocean include changes to precipitation and altered temperatures [12], [13], in concert with the influence of ocean acidification. Changes in rainfall will affect not only average salinities, but also the frequency and intensity of the most acute runoff events. These events lead to low-salinity extremes, requiring coastal and estuarine organisms to cope with rapid decreases in salinity. Adding complexity to these predicted changes is that areas that receive limited summer rainfall (e.g. Mediterranean climates) can experience hypersalinity and stratified water columns in estuaries and coastal areas; changes in thermal loading due to climate change could increase the frequency and duration of these stratification events [2], [14], [15].

Many emerging studies explore the effects of decreased pH in combination with simultaneous changes in another stressor [9], typically temperature but also salinity in a few studies [16], [17], [18]. By contrast, very little research exists to assess potential outcomes when multiple stressors are imposed sequentially instead of simultaneously; that is, when one stressor acts first by itself but is followed in rapid succession by another. Understanding how species respond to sequential stressors is particularly important in coastal and estuarine habitats. In these regions, organisms can experience sudden transitions from oceanic conditions to lower salinities due to episodic runoff events, or transitions to higher salinities due to vertical swimming through a stratified water column during mobile life stages. How future high-*p*CO_2_ conditions could affect an organism’s ability to cope with sudden changes in salinity has not yet been examined.

The porcelain crab *Petrolisthes cinctipes* serves as a useful model species for exploring such issues because it possesses life history characteristics representative of a variety of nearshore invertebrates that will experience changing hydrographic conditions in future decades. Although it occurs primarily in outer-coast intertidal mussel beds and cobble fields from Point Conception, California to the Queen Charlotte Islands, British Columbia [19], it also lives in estuaries where sufficient rocky habitat exists. Its congeners in the Atlantic are found most commonly in estuaries, where they associate with oyster beds and other biogenic habitat [20], [21]. Its planktotrophic larval stage remains relatively close to shore during its moderately long pelagic larval duration [22], making it similar to the larvae of many other invertebrates, including ecologically and commercially important taxa such as barnacles, mussels, oysters, and many other species of crabs [23], so the results of this study may be representative of how many other larvae will respond to future climate change.

Adults of many species in the genus *Petrolisthes* are relatively resilient to stressors such as desiccation [24], [25], high temperature [26], [27], [28], hypoxia [25], [29], and hyposaline conditions [30], which is evolutionarily consistent with the routine exposure of these organisms to the variable and stressful conditions of the intertidal zone and estuarine habitats. Though larval stages of many taxa are often more susceptible to stress than adults [31], two previous studies investigated the effects of elevated *p*CO_2_ in isolation on *P. cinctipes*, and both concluded that larvae were relatively resilient to *p*CO_2_ stress [32], [33]. These findings are consistent with the prediction that crustaceans will likely be one of the classes of organisms that are able to cope with future climate change [6], [34].

In this study, we determined how respiration by newly hatched larvae of the porcelain crab *Petrolisthes cinctipes* cultured under current (385 µatm) and future predicted (1000 µatm) levels of atmospheric *p*CO_2_ differed when exposed to low (22), normal (34), and high (40) salinity conditions. Though crustacean larvae are relatively good swimmers and can adjust their position in the water column to avoid unfavorable conditions, the mosaic of nearshore conditions results in larvae experiencing a range of salinities and pH. By culturing larvae under different *p*CO_2_ conditions and then exposing them to subsequent changes in salinity, we mimicked how larvae might encounter a plume of hyposaline runoff during their pelagic development or might experience hypersaline conditions when migrating through a stratified coastal embayment.

Oxygen consumption through respiration is a commonly used metric of stress in marine organisms, with stressful conditions usually resulting in a more rapid depletion of oxygen from the water, though metabolism could be suppressed under some conditions of stress [35]. Our investigation of how oxygen consumption differs across *p*CO_2_ and salinity treatments in this species therefore provides insights into how larval stages of nearshore invertebrates may respond to multiple, sequentially imposed stressors characteristic of future environmental conditions.

## Materials and Methods

Fifty ovigerous *Petrolisthes cinctipes* were collected from Shell Beach (38° 25.033′ N, 123° 06.350′ W) and Twin Coves (38° 27.490′ N, 123° 08.621′ W), California, using permits obtained from the California Department of Fish and Wildlife and California State Parks (no protected species were collected). Crabs were held in individual mesh containers in flow-through seawater at Bodega Marine Laboratory for 2–14 days. Containers were checked daily for hatched larvae, and newly released individuals from multiple broods (n = 24 broods) were transferred in mixed batches of 75 to replicate culture jars. These jars contained 0.45 um filtered seawater bubbled with prescribed *p*CO_2_ levels to maintain carbonate chemistry and water movement, and were held at a constant temperature and normal salinity (approximately 34) [36], [37]. At least five females contributed larvae to each culture jar, and broods were split evenly between *p*CO_2_ treatments. Not all females released larvae on the same night, which allowed culture start dates to be staggered and respirometry trials to be run on sequential days using larvae of the same age. The use of larvae from multiple broods helped to minimize brood effects that have been seen in other studies [32], [33], facilitating application of findings to the overall population.

Larvae were cultured at 14°C under two *p*CO_2_ conditions: 385 µatm (ambient) and 1000 µatm (elevated). The elevated *p*CO_2_ treatment, maintained by bubbling a premixed, NIST-traceable, air-CO_2_ gas mixture through the culture water, was selected to replicate global-average conditions projected for 2100 by fossil-fuel intensive climate model projections (A1FI scenario) [15], but also mimics transient conditions encountered even today [36]. Larvae were held at a density of 75 larvae per 3 L, and were fed newly hatched *Artemia* sp. nauplii at a density of 1 nauplius/ml [38]. The water in each culture jar was changed every two days by siphoning approximately 95% of the water from the jar and replacing it with new filtered seawater that had been equilibrated to the appropriate temperature and *p*CO_2_ treatment. During water changes, dead larvae and uneaten *Artemia* sp. were removed, and water exiting the jars was sampled for carbonate chemistry adhering to recommended practices (Table 1) [39]. We measured pH (total scale) [40] using a glass electrode (Accumet Excel XL60; Thermo Fisher Scientific, Waltham, Massachusetts, USA) and repeated analysis of certified TRIS reference material (measured in millivolts; batch no. 8; A. Dickson, Scripps Institute of Oceanography). Total alkalinity (TA) was measured by autotitration (Metrohm 809; Metrohm, Herisau, Switzerland), using the same certified reference material. Dissolved inorganic carbon (DIC) samples were processed using coulometric titration at the Monterey Bay Aquarium Research Institute (MBARI).

**Table 1:** Culturing system carbonate conditions.

CO_2_ Treatment	385 µatm	1000 µatm
pH (Total)	7.997±0.004	7.706±0.003
Ω Aragonite	2.033±0.019	1.127±0.007
Total Alkalinity (µmol/kg)	2243.5±5.1	2254.6±3.0
Dissolved Inorganic Carbon (µmol/kg)	2091.9±6.4	2178.3±4.0
*p*CO_2_ (µatm)	450.1±6.1	949.8±7.5

Mean values (± S. E.) of carbonate system conditions in larval culturing jars under ambient (385 µatm) and elevated (1000 µatm) *p*CO_2_ conditions. Larvae under ambient conditions experienced higher pH (p<0.0001) and lower DIC (p<0.0001) than those under elevated *p*CO_2_ conditions.

Respirometry trials were conducted in seawater at ambient *p*CO_2_ using a dissolved oxygen meter (model 781b Strathkelvin Instruments Ltd., Glasgow, UK) with a Clark-type microcathode polarographic electrode with a 22 micron diameter platinum cathode and silver/silver chloride anode connected by a buffered potassium chloride electrolyte solution (model 1302). We used a low permeability polypropylene membrane so the electrode could be used in solutions with minimal stirring, though we also visually checked to ensure the movement of larvae was sufficient to maintain water flow over the surface of the membrane. Data were transmitted via a data interface unit to a computer and the oxygen consumption rates were monitored using the Strathkelvin 949 Oxygen System (Version 2.2, Strathkelvin Instruments Ltd., Glasgow, UK).

**Figure 1:**
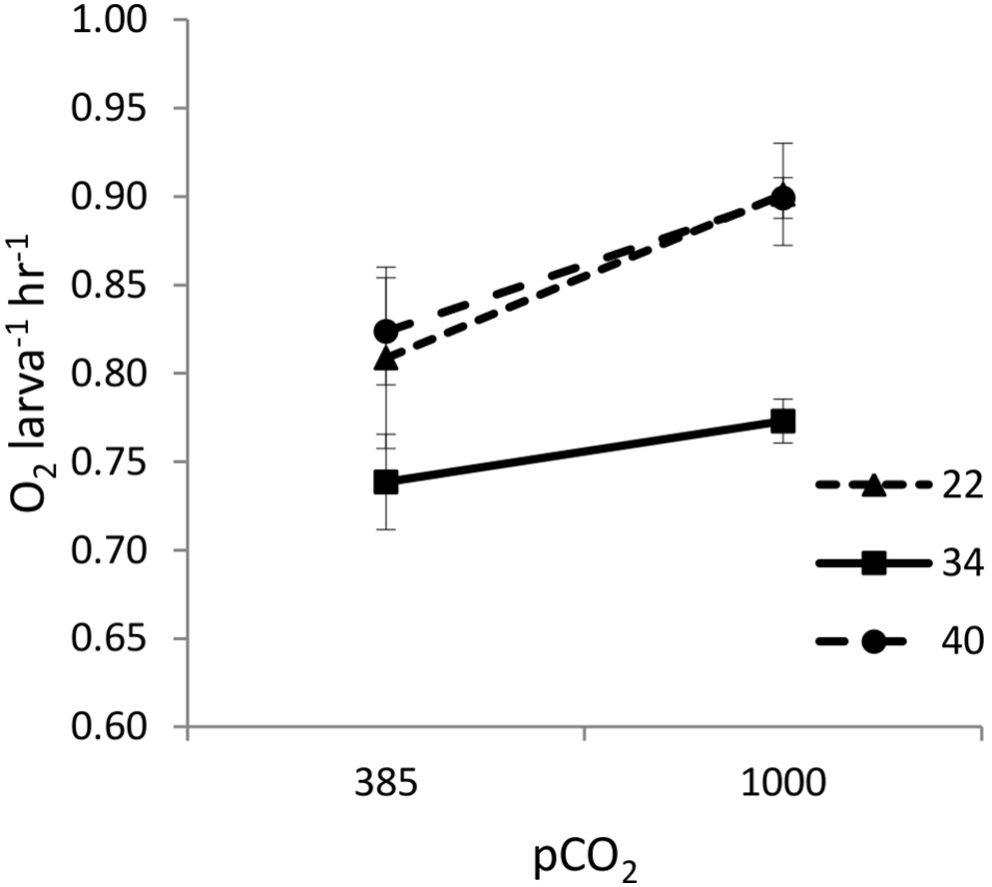
Larval oxygen consumption through time. Dissolved oxygen (mean total oxygen less background bacterial consumption rates, ± S.E.) during respirometry trials under ambient or dual-stressor conditions. Horizontal dashed line shows the lower limit of data analyzed in this study; data for oxygen levels under 5 mgL^−1^ are presented for completeness.

The electrode was placed in a PVC holder with double O rings to seal the chamber. The holder and electrode were inserted into a test chamber (26.34 mm×47.09 mm ID) holding 25 ml of test solution. To maintain the temperature at 14°C, this test chamber was jacketed in a dish (37.69 mm×75.30 mm ID) and kept in a constant temperature refrigeration unit. For each new trial, the electrode was calibrated using 2% sodium sulfite and air-saturated seawater to determine zero and saturated oxygen values, respectively. Before each series of experiments, a control chamber with no larvae was monitored to establish background microbial oxygen consumption rates, which were then subtracted from the values obtained during trial runs for our analyses. During each trial, eight first-stage larvae were removed from the culturing system and placed in the chamber for respirometry analysis, and visual analysis indicated that their activity was sufficient to maintain flow over the surface of the electrode. Seawater oxygen saturation state was measured every two minutes for one hour, though we only analyzed data from the start of the trial until the oxygen in the test chamber reached 5 mgL^−1^ to eliminate potential effects of hypoxia in the chamber on larval respiration (Figure 1). Trials with larvae from each *p*CO_2_ treatment were run sequentially in pairs (i.e., one trial with larvae from ambient *p*CO_2_ conditions was followed by one trial with larvae from elevated *p*CO_2_ conditions), and 13 pairs of trials were run at salinities of 34 over a period of 14 days.

Distilled water was added to filtered, ambient-*p*CO_2_ seawater (salinity 34) to make salinity 22 solution, and artificial seawater (Instant Ocean, Spectrum Brands, Inc.) was added to make salinity 40 solution. This procedure created two altered salinity treatments for testing effects of salinity stress on larvae drawn from each of our preceding *p*CO_2_ treatments. We acknowledge that these manipulated solutions have slightly different alkalinities and aragonite saturation states than natural seawater, and may have minor differences in trace elements [18]. Though we were unable to analyze carbonate system parameters of our test solutions during the experiment due to low volumes, we created solutions using the same methods to determine pH and total alkalinity for post-hoc estimates of alkalinity and aragonite saturation state. We also calculated these carbonate system parameters for hypothetical ocean waters of the same salinities as our treatments, using data collected in local coastal and estuarine waters [41].

Larvae were allowed to acclimatize in treatment solutions for ten minutes prior to respirometry trials to attenuate a startle response, and the system was calibrated before each trial. Ten minutes was sufficient for larvae to begin active swimming following their transfer. Paired trials (4 pairs at salinity 22 and 3 pairs at salinity 40) with larvae from each *p*CO_2_ treatment were run sequentially over a period of seven days.

We used ANOVAs to test for respiration rate differences between salinity treatments (ambient and stressful) and between *p*CO_2_ treatments (ambient and elevated), and Levene tests revealed our data met the assumption of equal variances. We used a Tukey HSD test to analyze differences among all four treatments. We also used ANOVAs to verify that our *p*CO_2_ treatments provided statistically different carbonate system conditions for developing larvae, and we used a Welch’s ANOVA for the single parameter (Ω aragonite) that did not meet the assumption of equal variances. All statistical analyses were conducted in JMP 9 (SAS Institute, Cary, NC).

## Results

Larvae experienced significantly different carbonate system conditions in our ambient and predicted future *p*CO_2_ treatments in the culturing apparatus (Table 1), including pH (ANOVA, F_1,173_ = 4451.7, p<0.0001), Ω aragonite (Welch’s ANOVA, F_1,87_ = 1925.2, p<0.0001), *p*CO_2_ (ANOVA, F_1,87_ = 2627.0, p<0.0001), and dissolved inorganic carbon (ANOVA, F_1,59_ = 137.0, p<0.0001). Analysis of altered-salinity solutions revealed that alkalinity and aragonite saturation state ranged from 1479–2880 µmol/kg and 0.63–2.16, respectively (Table 2). These values are extremely close to those estimated for natural coastal and estuarine waters in our area at those salinities (Table 2).

**Table 2:** Salinity treatment carbonate conditions.

		Treatment Solution	Predicted Natural Seawater
Salinity 22	pH	7.700	7.701
	*p*CO_2_	703.9	689.3
	Total Alkalinity (µmol/kg)	1479	1453
	Ω Aragonite	0.63	0.62
	Dissolved Inorganic Carbon (µmol/kg)	1445	1419
Salinity 40	pH	7.805	7.804
	*p*CO_2_	928.1	951.5
	Total Alkalinity (µmol/kg)	2880	2943
	Ω Aragonite	2.16	2.21
	Dissolved Inorganic Carbon (µmol/kg)	2707	2768

Calculated carbonate conditions of treatment solutions in which the salinity has been altered by adding distilled water (salinity 22) or Instant Ocean artificial seawater mix (salinity 40), compared to estimated carbonate conditions in natural coastal and estuarine waters of the same salinity [41].

**Figure 2:**
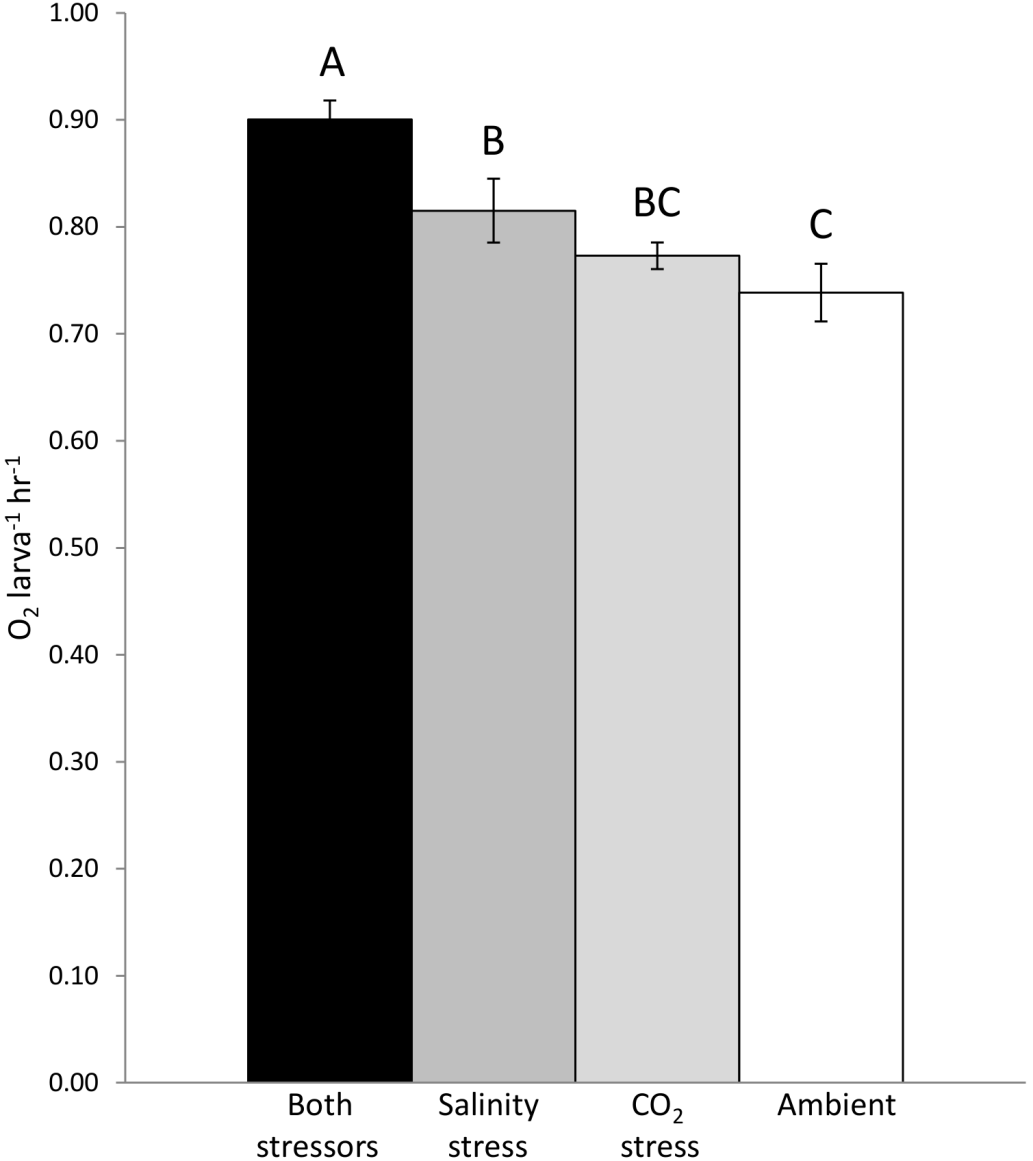
Multi-stressor effects on larvae. Oxygen consumption (mean ± S.E.) by larvae subjected to salinity stress, CO_2_ stress, both stressors, and ambient conditions; bars connected by the same letter are not significantly different. Larvae subjected to ambient conditions consumed significantly less oxygen than larvae subjected to both salinity and CO_2_ stress (p = 0.0149). There were no differences in oxygen consumption between larvae raised at ambient conditions and larvae subjected to a single stressor.

**Figure 3:**
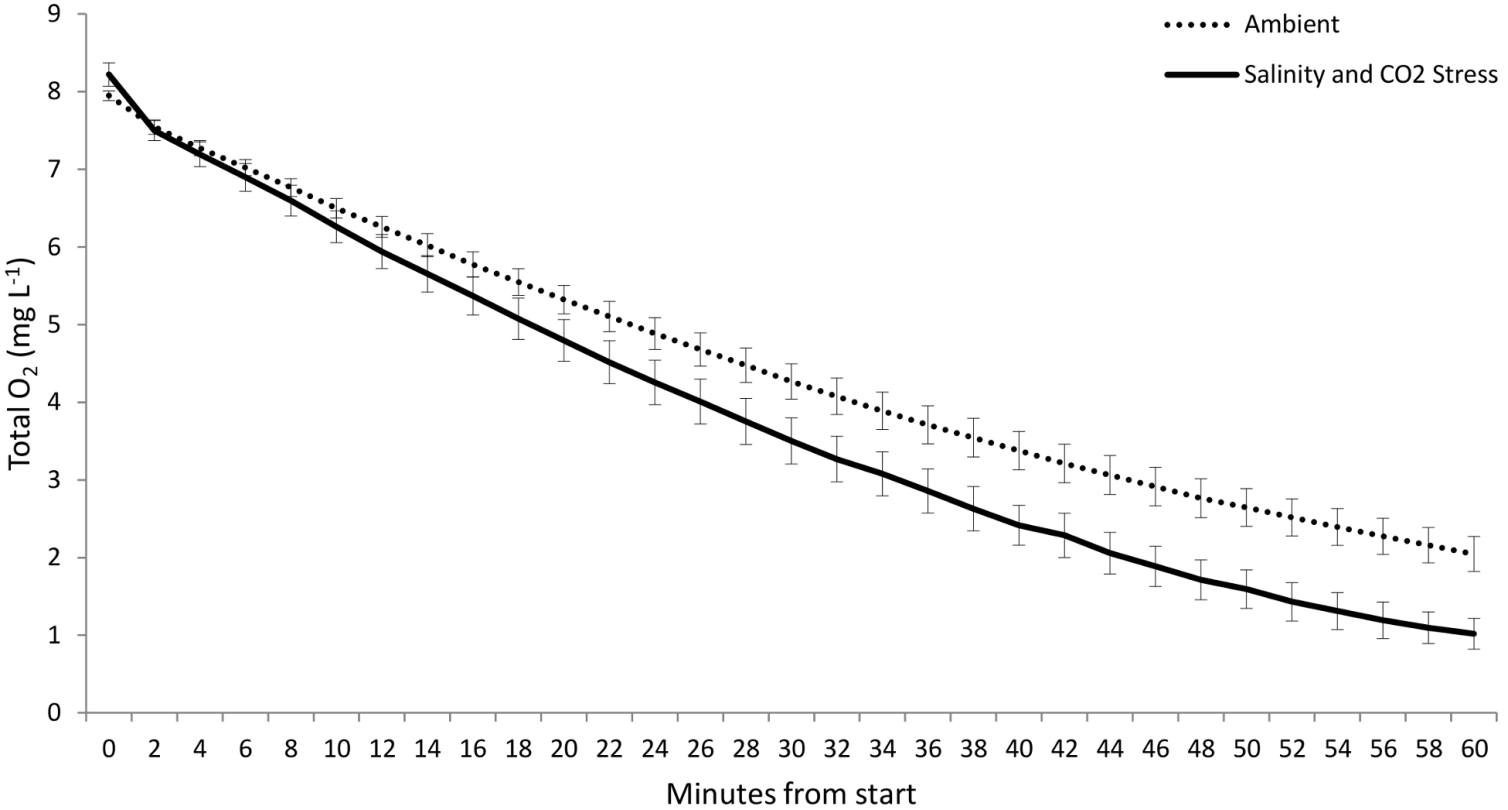
Respiration of larvae under stressful and ambient conditions. Oxygen consumption (mean ± S.E.) at stressful and ambient salinities by larvae raised under two *p*CO_2_ treatments. Overall, larvae consumed significantly less oxygen at ambient (34) salinity (open squares) than at depressed or elevated (22 or 40) salinities (filled squares, p = 0.0038), though there was no difference in oxygen consumption between *p*CO_2_ treatments (p = 0.1626).

There were no differences in oxygen consumption between larvae at ambient conditions and those exposed to a single stressor (Figure 2). Elevated *p*CO_2_ alone had no effect on larval oxygen consumption (Figure 3; F_1,40_ = 2.027, p = 0.1626), and larvae consumed less oxygen at ambient salinities than at stressful salinities (Figure 3; F_1,40_ = 9.529, p = 0.0038). Crab larvae raised under elevated *p*CO_2_ conditions that were subsequently exposed to high or low salinities consumed significantly more oxygen than larvae raised under ambient conditions and tested subsequently under normal salinities (Figure 2; F_3,40_ = 3.976, p = 0.0149).

## Discussion

Throughout our experiment, differences in carbonate chemistry existed not only between our *p*CO_2_ treatment conditions, but also among our salinity treatments used in the respirometry chamber. By moving larvae to solutions of altered salinities, alkalinities, and aragonite saturation states, we simulated the natural movement of larvae among water masses of varying physical characteristics in the field.

Crab larvae subjected to only a single stressor (salinity or CO_2_) showed no difference in oxygen consumption compared to larvae raised under ambient conditions (Figure 2), demonstrating that larvae in our study were resilient to a single stressor. However, larvae subjected to both stressors consumed significantly more oxygen than larvae raised under ambient conditions (Figure 2). This finding indicates that these larvae were negatively affected by sequentially imposed stressors, even though they were largely unaffected by single stressors. This has important implications for understanding how species will respond to the multiple stressors expected with future climate change.

Our finding that the combined future increases in *p*CO_2_ and changes to the hydrologic cycle could negatively affect crab larvae is somewhat surprising; most previous studies that have investigated the effect of future climate change on crustacean larvae have suggested that they will likely be largely unaffected [42], [43], [44], [45], [46], [47], including two previous studies on the same species used in our study [32], [33]. These studies, however, have primarily investigated single environmental stressors, or have imposed multiple stressors simultaneously. By applying multiple stressors sequentially in an environmentally relevant scenario that mimics realistic future conditions, we interpret that studies that investigate stressors individually might overestimate the resilience of crustacean larvae to future climate change. This investigation indicates that the timing of application of multiple stressors is an important factor to consider when studying the effects of future climate change. Not all stressors will act on the same timeframe, and some stressors such as changes in salinity may be sudden but temporary events.

Stressful conditions appear to negatively impact these organisms by increasing metabolic rates, which would increase food demands. This effect has implications for performance and survival, particularly during the larval phase, when individuals require energy not only for their daily metabolic needs, but also for the energetically costly process of metamorphosis–a final step before recruiting into benthic populations. Larvae with reduced energetic reserves have been shown to select poorer settlement sites [48] and have lower reproductive output as adults [49] than larvae that settle with larger reserves.

Stressors associated with future climate change also have been shown to impair the sensory abilities of multiple taxa in tropical systems [50], [51], [52] and in intertidal crustaceans in the Atlantic [53], [54]. Larvae of the genus *Petrolisthes* are gregarious settlers that rely on waterborne cues to find adult conspecifics that provide protection to newly settled postlarvae [55], [56], so if their sensory abilities are impaired by the metabolic stress documented in our study, it would have a negative impact on their settlement success.

Finally, although adults of the genus *Petrolisthes* are highly resilient to abiotic stressors found in the intertidal zone [25], [26], [27], [30], our work shows that larvae may be less resilient to stressors than previously believed, particularly when multiple stressors are imposed in succession, in a similar fashion to how they might be encountered by larvae swimming among water masses. This point indicates that even species that have evolved to live in abiotically stressful habitats such as the intertidal zone might be strongly affected by future climate change through susceptibilities manifesting during certain life stages.

## References

[1] Min S-K, Zhang X, Zwiers FW, Hegerl GC (2011) Human contribution to more-intense precipitation extremes. Nature 470: 378–381. 21331039 10.1038/nature09763

[2] Harley CDG, Hughes AR, Hultgren KM, Miner BG, Sorte CJB, et al (2006) The impacts of climate change in coastal marine systems. Ecology Letters 9: 228–241. 16958887 10.1111/j.1461-0248.2005.00871.x

[3] Karl TR, Trenberth KE (2003) Modern global climate change. Science 302: 1719–1723. 14657489 10.1126/science.1090228

[4] Pall P, Aina T, Stone DA, Stott PA, Nozawa T, et al (2011) Anthropogenic greenhouse gas contribution to flood risk in England and Wales in autumn 2000. Nature 470: 382–385. 21331040 10.1038/nature09762

[5] IPCC (2014) Chapter 5. Coastal Systems and Low-Lying Areas. In: Field CB, Barros VR, Dokken DJ, Mach KJ, Mastrandrea MD, et al., editors. Climate Change 2014: Impacts, Adaptation, and Vulnerability Part A: Global and Sectoral Aspects Contribution of Working Group II to the Fifth Assessment Report of the INtergovernmental Panel on Climate Change. Cambridge: Cambridge University Press.

[6] Kroeker KJ, Kordas RL, Crim RN, Singh GG (2010) Meta-analysis reveals negative yet variable effects of ocean acidification on marine organisms. Ecology Letters 13: 1419–1434. 20958904 10.1111/j.1461-0248.2010.01518.x

[7] Doney SC, Fabry VJ, Feely RA, Kleypas JA (2009) Ocean acidification: the other CO2 problem. Annual Review of Marine Science 1: 169–192. 10.1146/annurev.marine.010908.163834 21141034

[8] Kleypas JA, Buddemeier RW, Archer D, Gattuso J-P, Langdon C, et al (1999) Geochemical consequences of increased atmospheric carbon dioxide on coral reefs. Science 284: 118–143. 10102806 10.1126/science.284.5411.118

[9] Kroeker KJ, Kordas RL, Crim RN, Hendriks IE, Ramajo L, et al (2013) Impacts of ocean acidification on marine organisms: quantifying sensitivities and interaction with warming. Global Change Biology 19: 1884–1896. 23505245 10.1111/gcb.12179 PMC3664023

[10] Byrne M, Przeslawski R (2013) Multistressor impacts of warming and acidification of the ocean on marine invertebrates’ life histories. Integrative and Comparative Biology 53: 582–596. 23697893 10.1093/icb/ict049

[11] Najjar RG, Pyke CR, Adams MB, Breitburg D, Hershner C, et al (2010) Potential climate-change impacts on the Chesapeake Bay. Estuarine, Coastal and Shelf Science 86: 1–20.

[12] Diffenbaugh NS, Snyder MA, Sloan LC (2004) Could CO2-induced land-cover feedbacks alter near-shore upwelling regimes? Proceedings of the National Academy of Sciences 101: 27–32. 10.1073/pnas.0305746101 PMC314132 14691256

[13] Kueppers LM, Snyder MA, Sloan LC, Zavaleta ES, Fulfrost B (2005) Modeled regional climate change and California endemic oak ranges. Proceedings of the National Academy of Sciences 102: 16281–16286. 10.1073/pnas.0501427102 PMC1283413 16260750

[14] Di Lorenzo E, Miller AJ, Schneider N, McWilliams JC (2005) The warming of the California Current system: dynamics and ecosystem implications. Journal of Physical Oceanography 35: 336–362.

[15] IPCC (2007) Climate Change 2007, The Physical Science Basis. The Contribution of Working Group I to the Fourth Assessment Report of the Intergovernmental Panel on Climate Change. Cambridge: Cambridge University Press.

[16] Kaldy JE, Shafer DJ (2013) Effects of salinity on survival of the exotic seagrass *Zostera japonica* subjected to extreme high temperature stress. Botanica Marina 56: 75–82.

[17] Dickinson GH, Matoo OB, Tourek RT, Sokolova IM, Beniash E (2013) Environmental salinity modulates the effects of elevated CO2 levels on juvenile hard-shell clams, *Mercenaria mercenaria* . Journal of Experimental Biology 216: 2607–2618. 23531824 10.1242/jeb.082909

[18] Dickinson GH, Ivanina AV, Matoo OB, Portner HO, Lannig G, et al (2012) Interactive effects of salinity and elevated CO_2_ levels on juvenile eastern oysters, *Crassostrea virginica* . Journal of Experimental Biology 215: 29–43. 22162851 10.1242/jeb.061481

[19] Morris RH, Abbott DP, Haderlie EC (1980) Intertidal Invertebrates of California. Palo Alto, CA: Stanford University Press.

[20] Hollebone AL, Hay ME (2007) Population dynamics of the non-native crab Petrolisthes armatus invading the South Atlantic Bight at densities of thousands m^−2^ . Marine Ecology Progress Series 336: 211–223.

[21] Micheletti-Flores CV, Negreiros-Fransozo ML (1999) Porcellanid crabs (Crustacea, Decapoda) inhabiting sand reefs built by Phragmatopoma lapidosa (Polychaeta, Sabellariidae) at parapanapua beach, Sao Vicente, SP, Brazil. Revista Brasileira de Zoologia 59: 63–73.

[22] Morgan SG, Fisher JL, Miller SH, McAfee ST, Largier JL (2009) Nearshore larval retention in a region of strong upwelling and recruitment limitation. Ecology 90: 3489–3502. 20120816 10.1890/08-1550.1

[23] Shanks AL, Eckert GL (2005) Population persistence of California current fishes and benthic crustaceans: a marine drift paradox. Ecological Monographs 75: 505–524.

[24] Pellegrino CR (1984) The role of dessiccation pressures and surface area/volume relationships on seasonal zonation and size distribution of four intertidal decapod Crustacea from New Zealand: implications for adaptation to land. Crustaceana 47: 251–268.

[25] Lagos ME, Munoz JL, Contreras DA, Caceres CW (2011) Microhabitat segregation and physiological differences in two species of intertidal porcellanid crabs (Genus *Petrolisthes*) on the southern coast of Chile. Scientia Marina 75: 273–278.

[26] Stillman JH, Somero GN (1996) Adaptation to temperature stress and aerial exposure in congeneric species of intertidal porcelain crabs (genus *Petrolisthes*): correlation of physiology, biochemistry and morphology with vertical distribution. Journal of Experimental Biology 199: 1845–1855. 9319758 10.1242/jeb.199.8.1845

[27] Jensen GC, Armstrong DA (1991) Intertidal zonation among congeners: factors regulating distribution of porcelain crabs *Petrolisthes* spp. (Anomura: Porcellanidae). Marine Ecology Progress Series 73: 47–60.

[28] Stillman JH, Tagmount A (2009) Seasonal and latitudinal acclimatization of cardiac transcriptome responses to thermal stress in porcelain crabs, *Petrolisthes cinctipes* . Molecular Ecology 18: 4206–4226. 19765222 10.1111/j.1365-294X.2009.04354.x

[29] Yaikin J, Quinones RA, Gonzalez RR (2002) Aerobic respiration rate and anaerobic enzymatic activity of *Petrolisthes laevigatus* (Anomura, Porcellanidae) under laboratory conditions. Journal of Crustacean Biology 22: 345–352.

[30] Masunari S, Oliveira E, Kowalczuk VGL (1998) Decapod crustaceans from rocky shore at Farol Island, Matinhos, Parana, Brazil. Revista Brasileira de Zoologia 15: 219–239.

[31] Pechenik JA (1999) On the advantages and disadvantages of larval stages in benthic marine invertebrate life cycles. Marine Ecology Progress Series 177: 269–297.

[32] Carter HA, Ceballos-Osuna L, Miller NA, Stillman JH (2013) Impact of ocean acidification on metabolism and energetics during early life stages of the intertidal porcelain crab *Petrolisthes cinctipes* . Journal of Experimental Biology 216: 1412–1422. 23536589 10.1242/jeb.078162

[33] Ceballos-Osuna L, Carter HA, Miller NA, Stillman JH (2013) Effects of ocean acidification on early life-history stages of the intertidal porcelain crab *Petrolisthes cinctipes* . Journal of Experimental Biology 216: 1405–1411. 23536588 10.1242/jeb.078154

[34] Whiteley NM (2011) Physiological and ecological responses of crustaceans to ocean acidification. Marine Ecology Progress Series 430: 257–271.

[35] Sokolova IM (2013) Energy-limited tolerance to stress as a conceptual framework to integrate the effects of multiple stressors. Integrative and Comparative Biology 53: 597–608. 23615362 10.1093/icb/ict028

[36] Hettinger A, Sanford E, Hill TM, Russell AD, Sato KN, et al (2012) Persistent carry-over effects of planktonic exposure to ocean acidification in the Olympia oyster. Ecology 93: 2758–2768. 23431605 10.1890/12-0567.1

[37] Gaylord B, Hill TM, Sanford E, Lenz EA, Jacobs LA, et al (2011) Functional impacts of ocean acidification in an ecologically critical foundation species. The Journal of Experimental Biology 214: 2586–2594. 21753053 10.1242/jeb.055939

[38] Strathmann M (1987) Reproduction and development of marine invertebrates of the northern Pacific coast. Seattle: University of Washington Press.

[39] Dickson AG, Sabine CL, Shristian JR (2007) Guide to best practices for ocean CO_2_ measurements. PICES Special Publication 3: 1–191.

[40] Zeebe RE, Wolf-Gladrow D (2001) CO_2_ in seawater: equilibrium, kinetics, isotopes. Amsterdam: Elsevier.

[41] Smith SV, Hollibaugh JT (1997) Annual cycle and interannual variability of ecosystem metabolism in a temperate climate embayment. Ecological Monographs 67: 509–533.

[42] Donohue PJC, Calosi P, Bates AH, Laverock B, Rastrick S, et al (2012) Impact of exposure to elevated pCO_2_ on the physiology and behaviour of an important ecosystem engineer, the burrowing shrimp *Upogebia deltaura* . Aquatic Biology 15: 73–86.

[43] Long WC, Swiney KM, Foy RJ (2013) Effects of ocean acidification on the embryos and larvae of red king crab, *Paralithodes camtschaticus* . Marine Pollution Bulletin 69: 38–47. 23434384 10.1016/j.marpolbul.2013.01.011

[44] Styf HK, Skold HN, Eriksson SP (2013) Embryonic response to long-term exposure of the marine crustacean *Nephrops norvegicus* to ocean acidification and elevated temperature. Ecology and Evolution 3: 5055–5065. 24455136 10.1002/ece3.860 PMC3892368

[45] Walther K, Sartoris FJ, Bock C, Portner HO (2009) Impact of anthropogenic ocean acidification on thermal tolerance of the spider crab *Hyas araneus* . Biogeosciences 6: 2207–2215.

[46] McDonald MR, McClintock JB, Amsler CD, Rittschof D, Angus RA, et al (2009) Effects of ocean acidification over the life history of the barnacle *Amphibalanus amphitrite* . Marine Ecology Progress Series 385: 179–187.

[47] Pansch C, Nasrolahi A, Appelhans YS, Wahl M (2013) Tolerance of juvenile barnacles (*Amphibalanus improvisus*) to warming and elevated *p*CO_2_ . Marine Biology 160: 2023–2035.

[48] Burgess SC, Treml EA, Marshall DJ (2012) How do dispersal costs and habitat selection influence realized population connectivity? Ecology 93: 1378–1387. 22834378 10.1890/11-1656.1

[49] Burgess SC, Marshall DJ (2011) Are numbers enough? Colonizer phenotype and abundance interact to affect population dynamics. Journal of Animal Ecology 80: 681–687. 21250991 10.1111/j.1365-2656.2010.01802.x

[50] Munday PL, Dixson DL, McCormick MI, Meekan M, Ferrari MCO, et al (2010) Replenishment of fish populations is threatened by ocean acidification. Proceedings of the National Academy of Sciences 107: 12930–12934. 10.1073/pnas.1004519107 PMC2919925 20615968

[51] Doropoulos C, Ward S, Diaz-Pulido G, Hoegh-Guldberg O, Mumby PJ (2012) Ocean acidification reduces coral recruitment by disrupting intimate larval-algal settlement interactions. Ecology Letters 15: 338–346. 22321314 10.1111/j.1461-0248.2012.01743.x

[52] Ferrari MCO, McCormick MI, Munday PL, Meekan M, Dixson DL, et al (2012) Effects of ocean acidification on visual risk assessment in coral reef fishes. Functional Ecology 26: 553–558.

[53] de la Haye KL, Spicer JI, Widdicombe S, Briffa M (2012) Reduced pH sea water disrupts chemo-responsive behaviour in an intertidal crustacean. Journal of Experimental Marine Biology and Ecology 412: 134–140.

[54] de la Haye KL, Spicer JI, Widdicombe S, Briffa M (2011) Reduced sea water pH disrupts resource assessment and decision making in the hermit crab *Pagurus bernhardus* . Animal Behaviour 82: 495–501.

[55] Jensen GC (1991) Competency, settling behavior, and postsettlement aggregation by porcelain crab megalopae (Anomura: Porcellanidae). Journal of Experimental Marine Biology and Ecology 153: 49–61.

[56] Jensen GC (1989) Gregarious settlement by megalopae of the porcelain crabs *Petrolisthes cinctipes* (Randall) and *P. eriomerus* Stimpson. Journal of Experimental Marine Biology and Ecology 131: 223–231.

